# Supplementary material for: Quantification and Profiling of Early and Late Differentiation Stage T Cells in Mantle Cell Lymphoma Reveals Immunotherapeutic Targets in Subsets of Patients
Source: Cancers (Basel). 2024 Jun 21;16(13):2289. doi: 10.3390/cancers16132289 (PMC11240320; doi:10.3390/cancers16132289)
Supplement: Supplementary file 1 [file cancers-16-02289-s001.zip › cancers-3017666-Supplementary File S1.pdf]

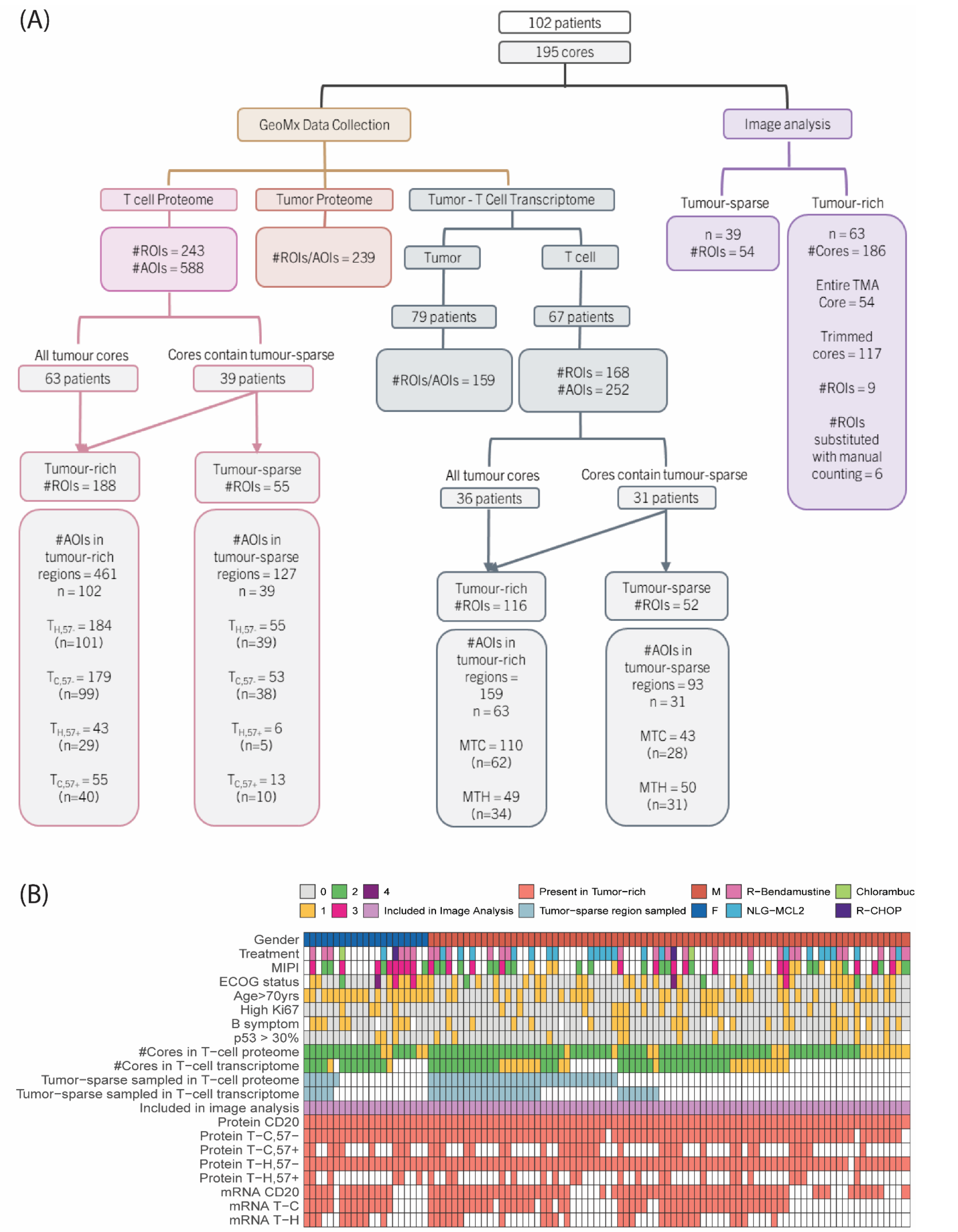

Figure S1: Overview of data collection: A) Summarized information of the number of patients, patient cores, spatially-guided region of interest (ROIs) and cell-specific segments collected by GeoMx™ DSP. B) Tile plot showing the distribution of technical and clinical parameters across the studied patients (n=102). For most patients two cores were included in the study. In tumor sparse (TS) regions T-cell proteomic and transcriptomic data was collected from 39 and 31 patients, respectively.

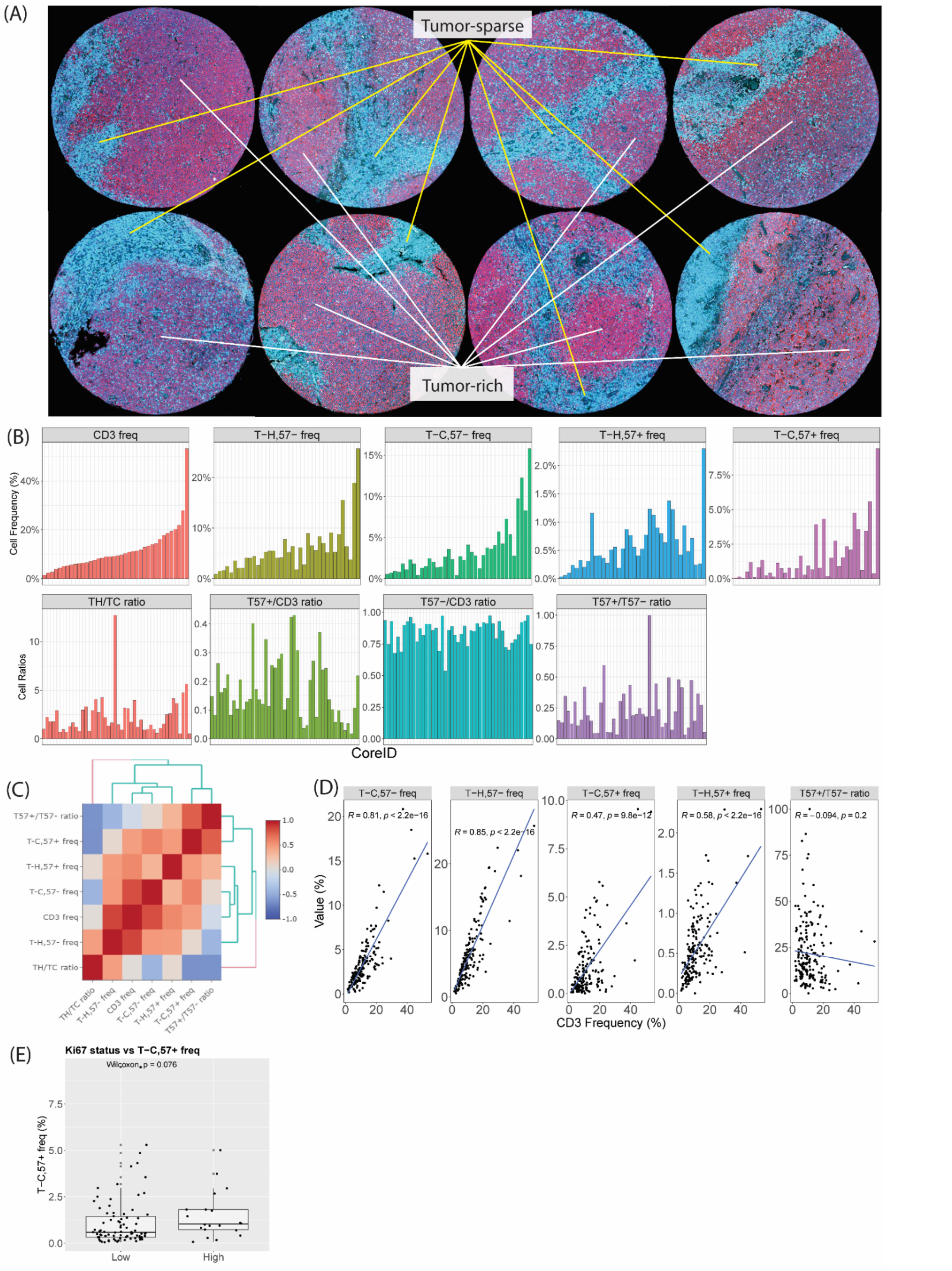

Figure S2: Composition of MCL TIME based on image analysis metrics (n=102): A) Examples of tissue micro array cores containing both tumor-sparse and rich areas, stained with CD3 (cyan), CD20 (red) and Syto13 (blue, nuclei marker). B) Distribution of the various infiltrating frequencies and ratios with respect to each core. X-axis is ordered

as per increasing CD3 infiltration shown in the top panel, first plot. C) Spearman correlation analysis between the T-cell infiltrating frequencies and ratios. D) Spearman correlation between infiltrating CD3 frequency vs T_{57-} and T_{57+} frequency as well as T_{57+}/T_{57-} ratio. E) Boxplot analysis of T_{57+} frequency in Ki-67 high and low subgroups (based on IHC staining).

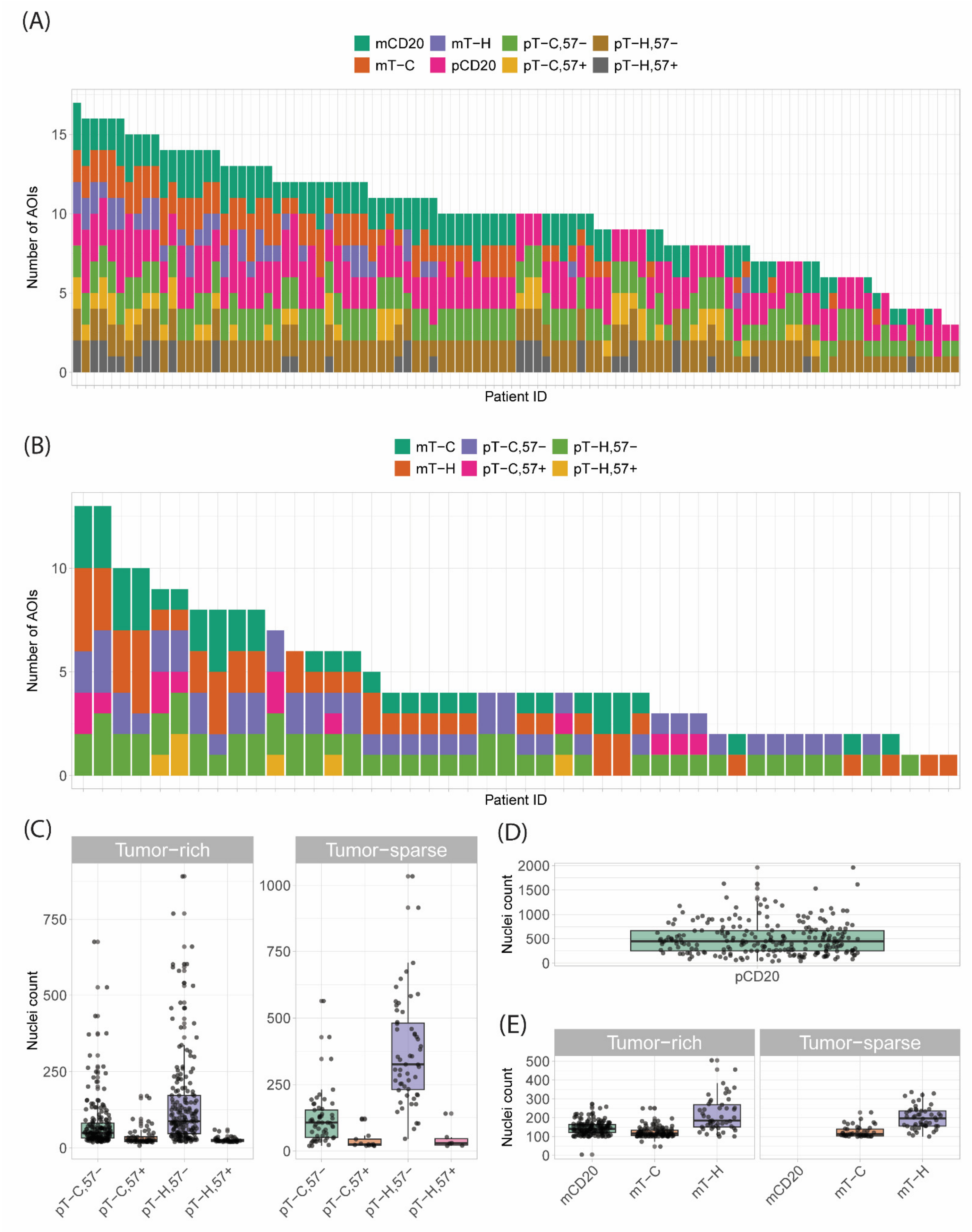

Figure S3: Distribution of segments and nuclei count as collected for each omic data set: A) Barplot of number of segments collected per patient per omic layer for the tumor-rich compartment. B) Barplot of number of segments

collected per patient per omic data set for the tumor-sparse compartment. Boxplot distribution of the nuclei count for C) T-cell proteome in tumor-sparse and -rich regions, D) CD20 proteome, E) CD20 and T-cell transcriptome in tumor-rich and -sparse regions.

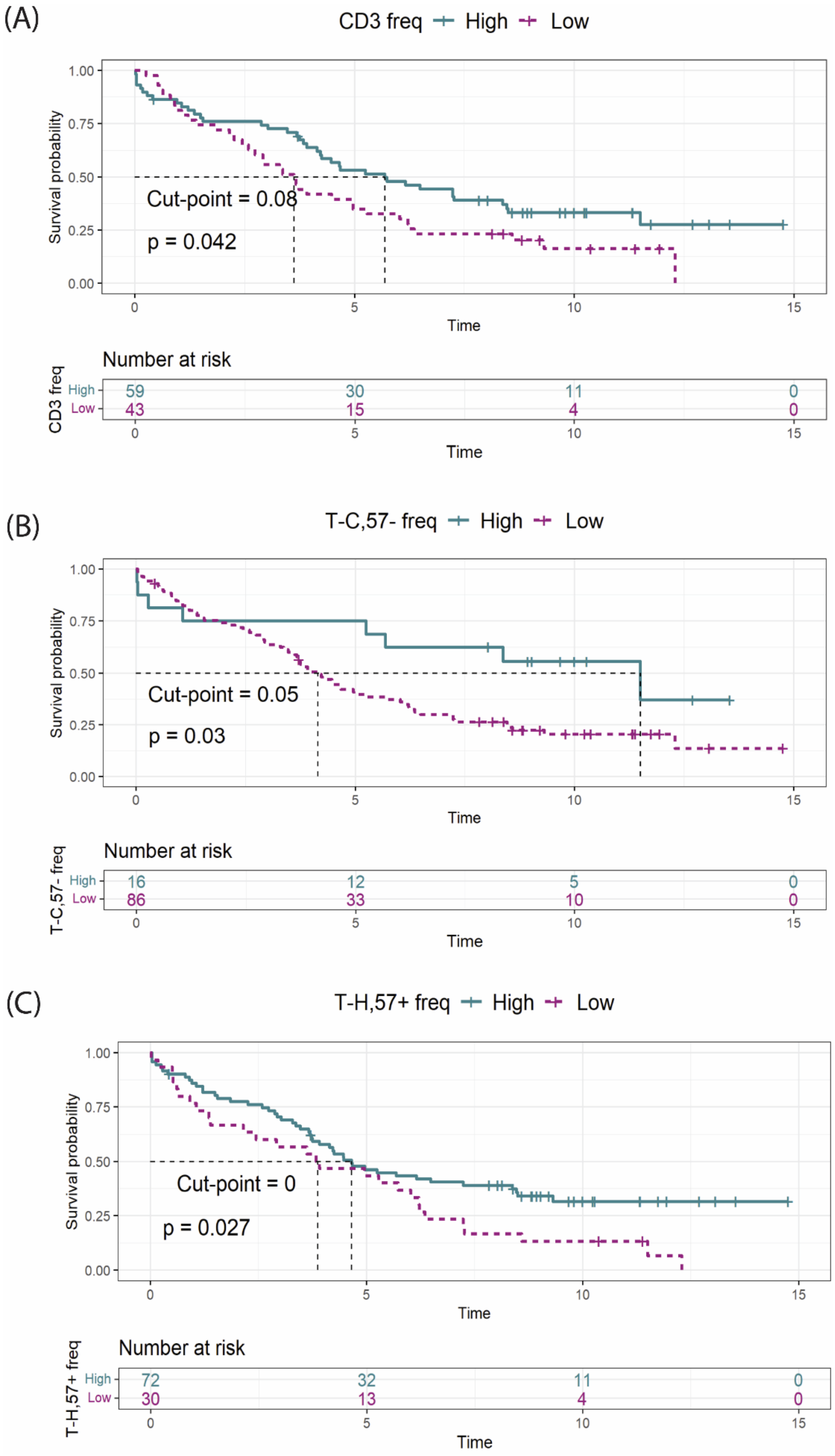

Figure S4: Outcome analysis based on infiltrating T-cell frequencies (n=102): A) Kaplan-Meier and log-rank analysis of A) CD3, B) $T_{C,57-}$, and C) $T_{H,57+}$ frequencies with stated cut-off (%). The groups were defined by max-rank statistics with minimum population distribution set at 0.1 (10%).

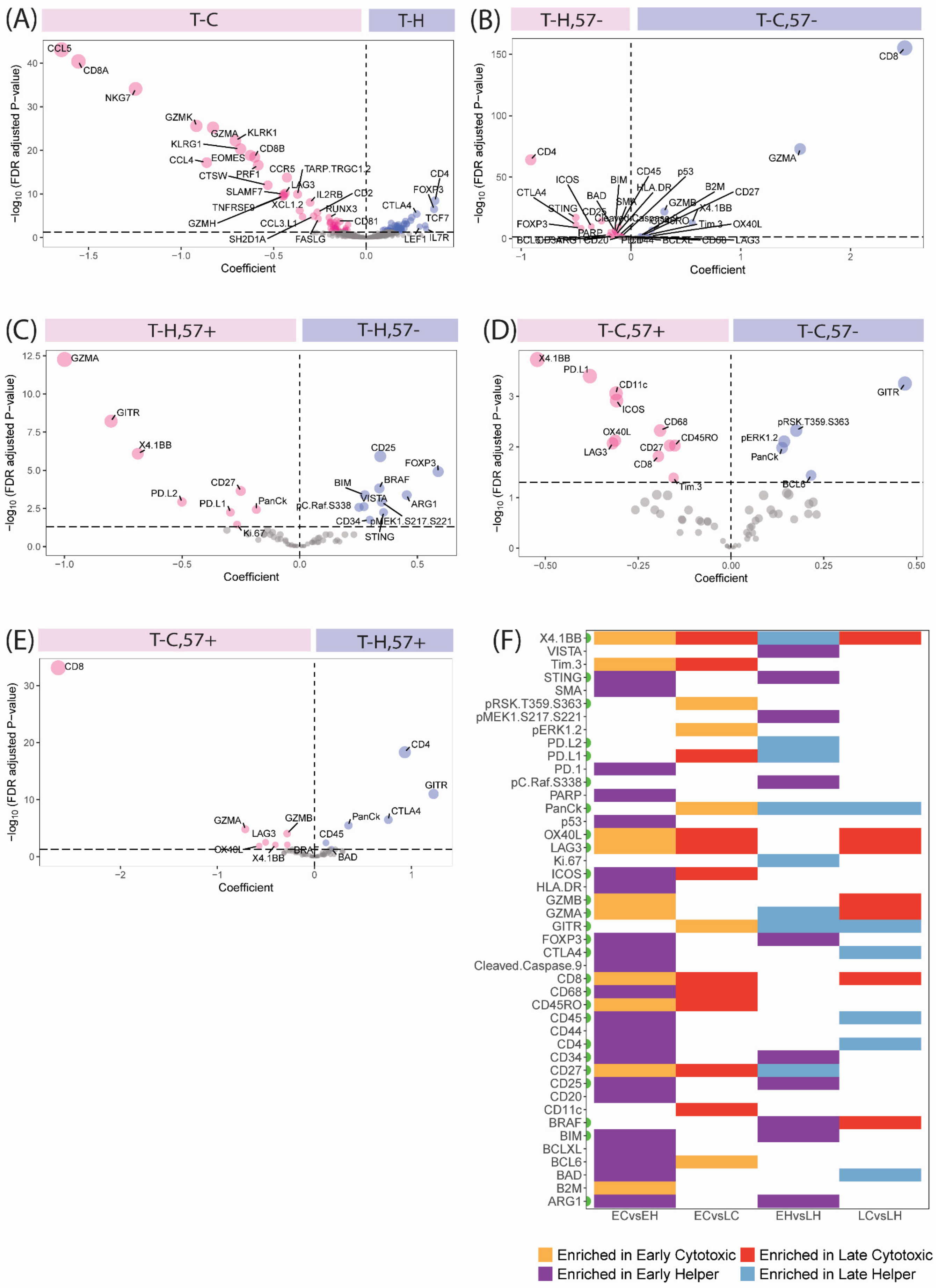

Figure S5: Differential expression analysis of infiltrating T-cell subsets: Linear mixed model analysis highlighting (A) differentially expressed transcripts in T_H vs T_C and differentially expressed proteins in B) $T_{C,57-}$ vs $T_{H,57-}$, C) $T_{H,57-}$ vs $T_{H,57+}$, D) $T_{C,57-}$ vs $T_{C,57+}$ and E) $T_{H,57+}$ vs $T_{C,57+}$. F) Tile plot summarizing the differentially expressed proteins identified in B-F

highlighting the direction of enrichment of proteins in the four subtypes. The green dots on the y-label highlight the proteins identified by ANOVA as shown in figure 6.

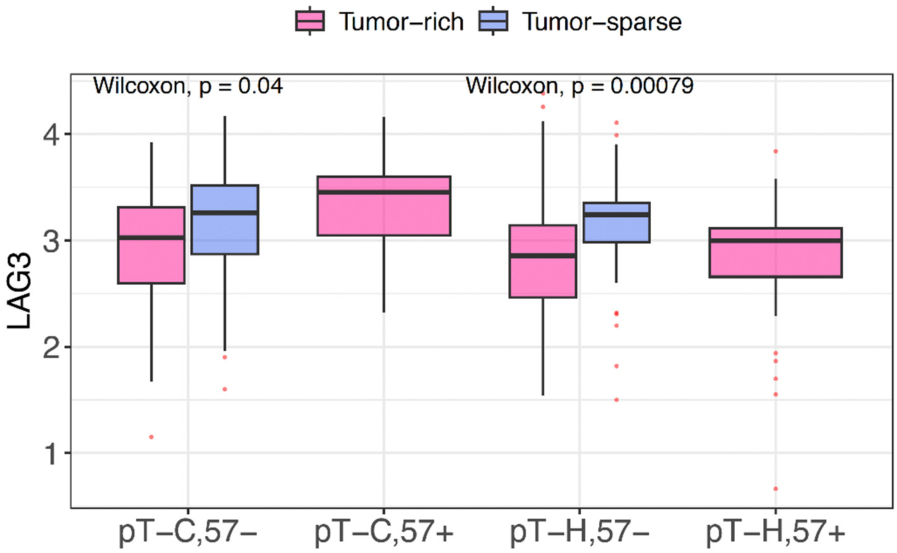

Figure S6: Boxplot analysis of LAG3 protein expression. Expression plotted for the four T-cell subsets across the various regions, indicating that $T_{C,57+}$ T cells show the highest mean expression of LAG3 in tumor-rich microenvironment.

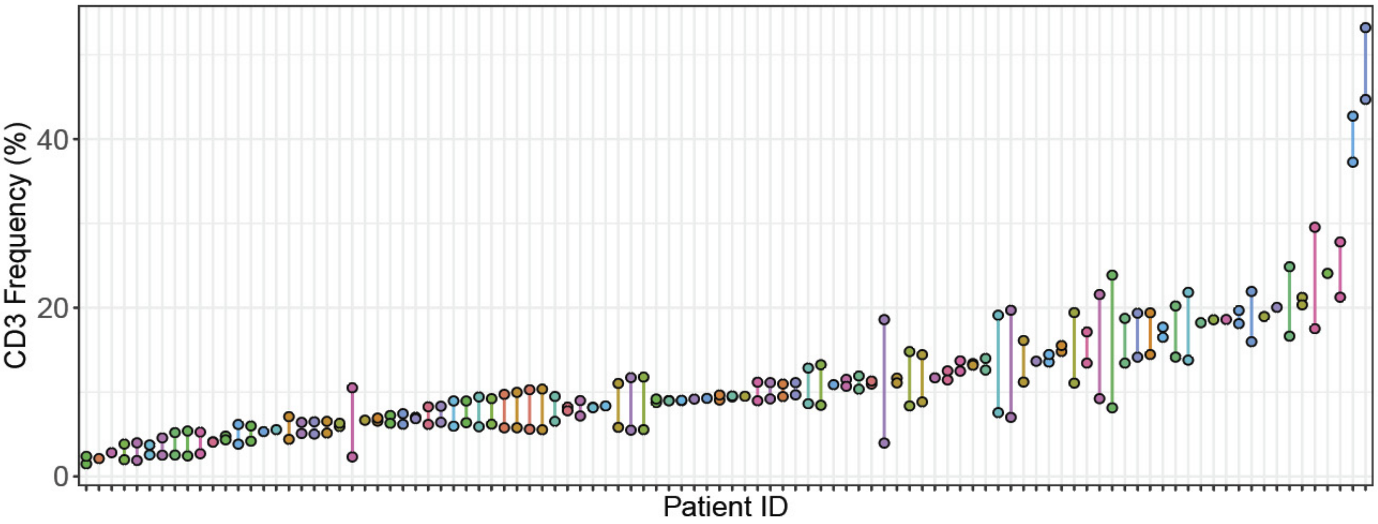

Figure S7: Exploration of CD3 infiltration between the duplicate cores per patient (n=102). Plot shows that the duplicate cores have acceptable variation to allow the use of aggregated means in subsequent analysis.

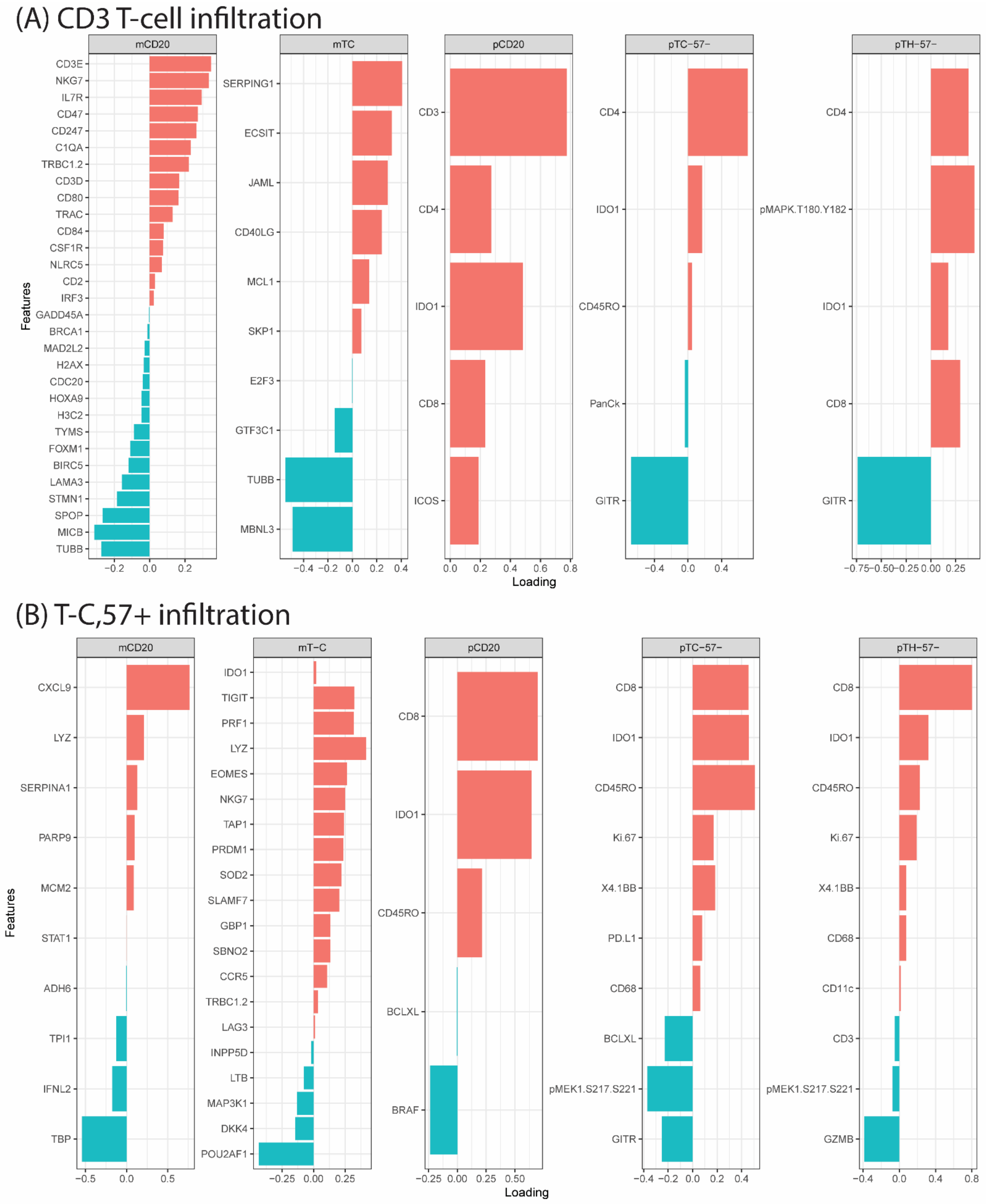

Figure S8: Multi-omics integration with respect to CD3 and $T_{C,57+}$ infiltration (n=62). Bar plot of estimated loadings of omics-layer specific features for component-1 as identified by DIABLO data integration modelling for infiltration groups of A) CD3 and B) $T_{C,57+}$ frequency.

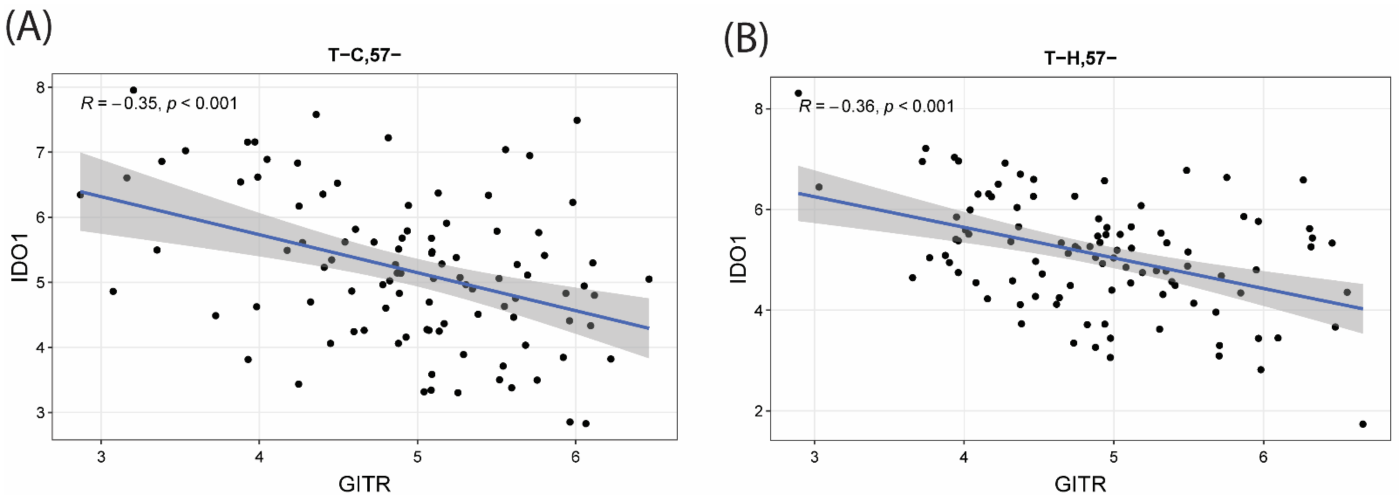

Figure S9: Spearman correlation of IDO1 – GITR (n=102). Analysis performed for infiltrating (tumor-rich) A) $T_{C,57-}$ and B) $T_{H,57-}$, showing significant negative association between the two markers.
